# Myopericytoma in urinary bladder: a case report

**DOI:** 10.1186/s13256-017-1226-2

**Published:** 2017-02-19

**Authors:** Takahiro Nagai, Toshio Kamimura, Kaoru Itou, Masato Fujii, Hiromasa Tsukino, Shoichiro Mukai, Yutaka Akiyama, Hiroaki Kataoka, Toshiyuki Kamoto

**Affiliations:** 1 Department of Urology, Faculty of Medicine, University of Miyazaki, 5200 Kihara, Kiyotake, Miyazaki, Japan; 2 Section of Oncopathology and Regenerative Biology, Faculty of Medicine, University of Miyazaki, 5200 Kihara, Kiyotake, Miyazaki, Japan

**Keywords:** Myopericytoma, Bladder tumor, Submucosal tumor

## Abstract

**Background:**

Myopericytoma is reported to occur mainly in the skin and superficial soft tissue of the extremities. In contrast, occurrence in the urinary bladder is extremely rare.

**Case presentation:**

We describe a 75-year-old Japanese man who developed a submucosal tumor at the right trigone of his bladder that led to interference with the discharge of right ureteral calculus. No invasive growth was observed by magnetic resonance imaging. Transurethral resection was successfully performed; histopathological analysis revealed perivascular proliferation of spindle-shaped to oval-shaped, cytologically bland tumor cells with eosinophilic cytoplasm. On immunohistochemical examination, the tumor cells were positive for alpha-smooth muscle actin, desmin, CD34 and *h*-caldesmon.

**Conclusion:**

Cystoscopic and pathological findings were compatible with a diagnosis of myopericytoma of the urinary bladder.

## Background

Mesenchymal tumor in the bladder accounts for less than 1 % of all bladder neoplasms [1]. Although benign mesenchymal tumors are also rare, leiomyoma is the most common, followed by hemangioma, solitary fibrous tumor, and neurofibroma [1–3].

Myopericytoma is a benign mesenchymal neoplasm composed of cytologically uniform presentation ranging from oval to spindle-shaped cells with a myoid appearance showing multilayered, perivascular concentric growth [2, 3]. Myopericytoma is reported to occur typically in skin and superficial soft tissue, commonly in the distal and proximal extremities, and occasionally in the trunk, head, and neck. In all locations the mass is solitary, painless, and slow growing [2–10]. In rare cases, the tumor is found in the oral cavity, nasal cavity, and thorax [2–10]. By contrast, occurrence in visceral organs such as the lungs, heart, gastrointestinal tract, and brain is extremely rare [2–10]. Furthermore, only 11 cases (ten cases in the kidney and one case in urinary bladder) in the urinary tract have been described [4–10].

In the present case report, we review our experience with a case of myopericytoma occurring in urinary bladder, and report the clinicopathological features.

## Case presentation

**Fig. 1:**
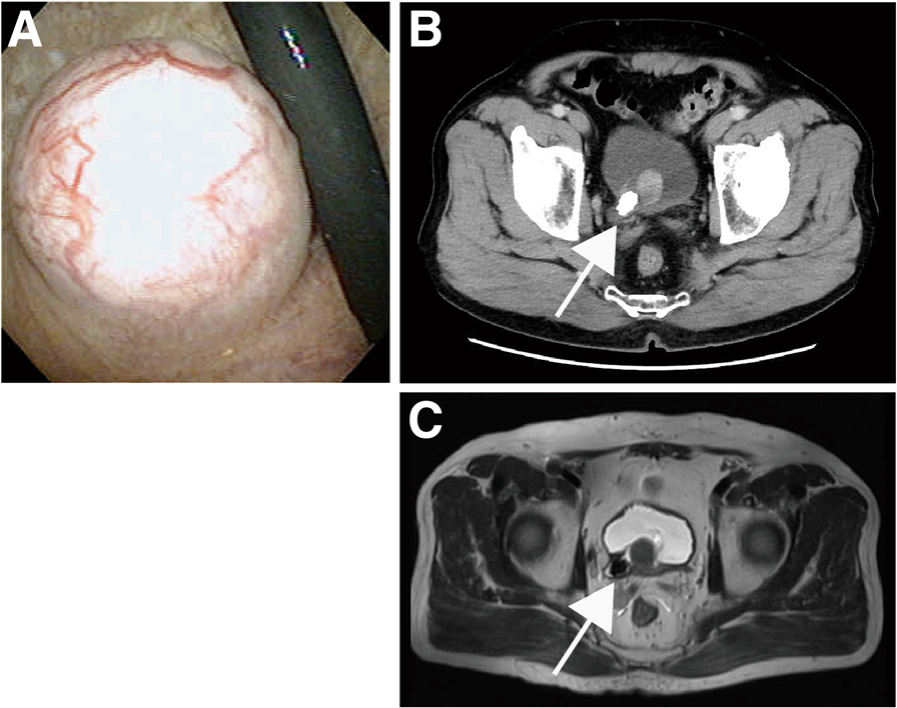
**a** Cystoscopy revealed a submucosal tumor covered with normal epithelium. **b** Enhanced computed tomography showed right ureteral calculus (*arrow*) and the tumor with enhancement. **c** Magnetic resonance imaging (T2-weighted image) revealed right ureteral calculus (*arrow*) and tumor

A 75-year-old Japanese man was referred for sudden asymptomatic gross hematuria. Ultrasonography and abdominal X-ray suggested bladder calculus, and computed tomography (CT) was performed. CT showed intramural right ureteral calculus (1.8 cm in diameter) and a well-circumscribed hypervascular mass measuring approximately 2.1 cm at the right trigone of his urinary bladder (Fig. 1b). Hydronephrosis was absent in bilateral kidneys. We then performed a cystoscopy to confirm the bladder mass. Examination revealed a non-papillary nodular mass located at the right trigone (Fig. 1a). The mass appeared to be completely covered by normal urothelium, suggesting a submucosal tumor. The mucosa was elevated behind the mass; however, we were unable to confirm the right ureteral orifice and calculus preoperatively. Since CT showed the mass to be adjacent to the calculus, the tumor may have interfered with the discharge of the calculus. No invasive growth was observed by magnetic resonance imaging (Fig. 1c). Urine cytology was negative for malignant cells. We then performed transurethral resection (TUR) of the mass. Following resection, we confirmed right ureteral orifice and intra-ureteral calculus. As a result, we performed transurethral lithotripsy. Our patient recovered without perioperative complications and has remained free of recurrence for 5 months.

### Pathological features

On gross examination, the resected surface of the mass specimens was solid, firm, and tan-white to yellow without apparent necrosis and hemorrhage.

**Fig. 2:**
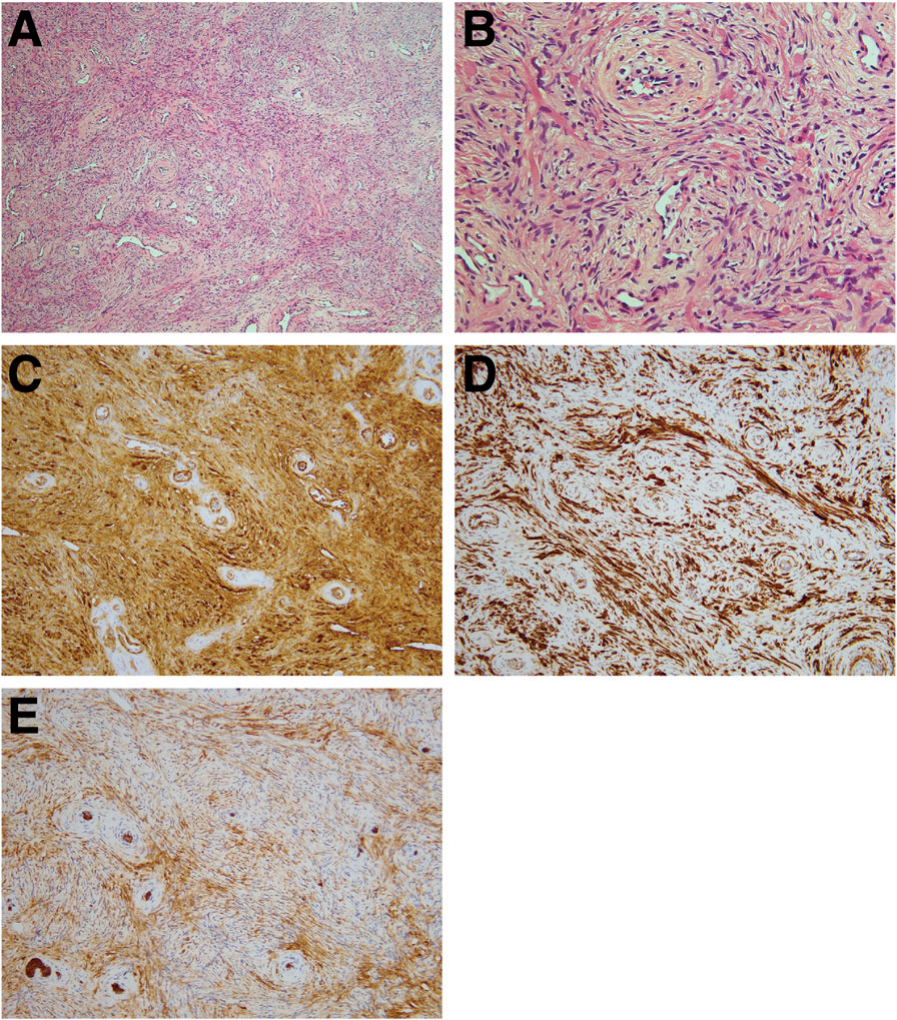
Histopathologic findings of the tumor. Hematoxylin and eosin staining. Low magnification (**a**) (×100) and high magnification (**b**) (×400) show the concentric perivascular proliferation of spindle-shaped cells with eosinophilic cytoplasm and bland nuclei. The tumor cells revealed positive reactivity for smooth muscle actin (**c**) (×200), caldesmon (**d**) (×200), and CD34 (**e**) (×200). Endothelial cells are also positive for CD34 staining as well as the tumor cells (**e**)

On histologic examination, the urothelium was largely denuded; however, residual urothelium showed no atypia. In the submucosal lesion, we observed spindle-shaped to oval-shaped cells that had mildly hyperchromatic nuclei and eosinophilic elongated or tapering cytoplasm arranged in loose fascicles or perivascular whorls around often-hyalinized blood vessels or in a vague storiform-growth fashion, embedded in variably fibromyxoid or edematous stroma associated with dilated thin-walled blood vessels (Fig. 2a, b). Mitotic figures are rare. On immunohistochemical examination, the tumor cells were positive for alpha-smooth muscle actin (SMA), desmin, CD34, and *h*-caldesmon, and CD99 was weakly and focally expressed (Fig. 2c-e). The cells were negative for S-100, CAM5.2, AE1/AE3, epithelial membrane antigen (EMA), STAT6, MUC4, and claudin-1.

## Discussion

Myopericytoma is defined as a member of the perivascular tumor family, which includes myofibroma, angioleiomyoma, glomus tumor, and myopericytoma, by the World Health Organization (WHO) classification (of tumor of soft tissue and bone) [2]. Myopericytoma occurs in various age groups, reported as ranging from 10 to 87 years; however, most are seen in adults with a median age of 49 years [3]. Regarding gender, a slight male predominance was reported in a male to female ratio of 1.25:1 [3]. As mentioned above, the tumor generally arises in dermal and subcutaneous tissue, and occurrence in visceral or intracranial sites is extremely rare [2–10]. To the best to our knowledge, the current case is the second report in the English literature [5]. Compared with a previous report by Zhao *et al.* [5], the majority of clinical features are similar, except for gross hematuria. In our case, the position of the tumor relative to the ureteral calculus was very unusual. Because the tumor was located immediately adjacent to the right ureteral orifice, it may have interfered with the discharge of the calculus. We speculated that the gross hematuria might have been the result of mucosal injury caused by the calculus. However, the results of cystoscopic and enhanced CT were nearly identical in both cases.

On histopathologic examination, morphological features were consistent with myopericytoma. On immunohistochemical examination, the majority of the findings were also consistent; however, unusual intense immunoreactivity was observed in CD34. Although, myopericytoma is reported to be negative for CD34 in most cases, one case of strong and three cases of partial reactivity have been reported in the kidney [6, 7, 9].

Myopericytoma shares several morphologic and immunohistochemical features with myofibroma, angioleiomyoma, and glomus tumor [2, 3]. However, each tumor has distinct characteristics. Myofibroma is characterized by a multinodular and biphasic growth pattern of peripherally located mature spindle-shaped myoid cells arranged in bundles, and centrally located immature-appearing oval-shaped tumor cells associated with branching thin-walled vessels, which are the most significant appearance for differential diagnosis [2, 7]. Angioleiomyoma shares the appearance of concentric perivascular growth pattern with myopericytoma; however, angioleiomyoma tends to lack the distinctive thick-walled vessels and shows negative or focal reactivity for desmin immunohistochemically [2, 7].

Myopericytoma is generally considered a benign tumor with favorable clinical course, although local recurrence has been reported in a small number of cases [3–10]. However, a few cases of malignant myopericytoma have been reported [3–10]. The proposed pathological criteria for malignancy are reported as deep-seated location, infiltrative growth pattern, nuclear pleomorphism, high mitotic activity, and presence of tumor necrosis [7, 9]. In these reports, six of eight patients with malignant myopericytoma developed metastasis, and three patients died of the disease within 1 year [9]. The majority of these malignant tumors occurred in extremities, and one case each of neck, superior mediastinum, and left atrium were involved [9]. No recurrence or metastasis has been reported in cases occurring in the urinary tract [9]. In our case, cystoscopy and CT showed that the patient has remained free from recurrence for 5 months.

## Conclusions

While myopericytoma commonly occurs in skin and superficial soft tissue, it is extremely rare in the urinary bladder. We treated a 75-year-old Japanese man with submucosal tumor adjacent to the right ureteral orifice. The tumor may have interfered with the discharge of ureteral calculus. Cystoscopic and pathological findings prompted the conclusion that the tumor was the urinary bladder counterpart of myopericytoma.

## Abbreviations

CT: Computed tomography
EMA: Epithelial membrane antigen
SMA: Smooth muscle actin
TUR: Transurethral resection
WHO: World Health Organization

## References

[1] Lott S, Lopez-Beltran A, Maclennan GT, Montironi R, Cheng L. Soft tissue tumors of the urinary bladder, Part I: myofibroblastic proliferations, benign neoplasms, and tumors of uncertain malignant potential. Hum Pathol. 2007;38(6):807–23. 10.1016/j.humpath.2007.03.017 17509394

[2] Fletcher CD, Bridge JA, Hogendoom PC, Mertens F. World Health Organization classification of tumours of soft tissue and bone. Lyon: IARC Press; 2013.

[3] Samaratunga H, Delahunt B. Mesenchymal tumors of adult kidney. Semin Diagn Pathol. 2015;32(2):160–71. 10.1053/j.semdp.2015.02.007 25773128

[4] Rodríguez D, Cornejo KM, Sadow PM, Santiago-Lastra Y, Feldman AS. Myopericytoma tumor of the glans penis. Can J Urol. 2015;22(3):7830–3. 26068635

[5] Zhao M, Williamson SR, Sun K, Zhu Y, Li C, Xia W, Qi H, Wang L, Linos K, Cheng L. Benign perivascular myoid cell tumor (myopericytoma) of the urinary tract: a report of 2 cases with an emphasis on differential diagnosis. Hum Pathol. 2014;45(5):1115–21. 10.1016/j.humpath.2013.12.017 24746217

[6] Lau SK, Klein R, Jiang Z, Weiss LM, Chu PG. Myopericytoma of the kidney. Hum Pathol. 2010;41(10):1500–4. 10.1016/j.humpath.2010.02.014 20655090

[7] Dhingra S, Ayala A, Chai H, Moreno V, Zhao B. Renal myopericytoma: case report and review of literature. Arch Pathol Lab Med. 2012;136(5):563–6. 10.5858/arpa.2011-0387-CR 22540305

[8] Zhang Z, Yu D, Shi H, Xie D. Renal myopericytoma: A case report with a literature review. Oncol Lett. 2014;7(1):285–7. 10.3892/ol.2013.1678 PMC3861592 24348865

[9] Li J, Zhao M, Chen Z, Zou L, Teng X. Renal myopericytoma: a clinicopathologic study of six cases and review of the literature. Int J Clin Exp Pathol. 2015;8(5):4307–20. PMC4502995 26191123

[10] Mentzel T, Dei Tos AP, Sapi Z, Kutzner H. Myopericytoma of skin and soft tissues: clinicopathologic and immunohistochemical study of 54 cases. Am J Surg Pathol. 2006;30:104–13. 10.1097/01.pas.0000178091.54147.b1 16330949

